# Air-Stable and Eco-Friendly Symmetrical Imine with Thiadiazole Moieties in Neutral and Protonated form for Perovskite Photovoltaics

**DOI:** 10.3390/ma17081909

**Published:** 2024-04-20

**Authors:** Krzysztof Artur Bogdanowicz, Agnieszka Iwan, Karolina Dysz, Wojciech Przybyl, Monika Marzec, Kacper Cichy, Konrad Świerczek

**Affiliations:** 1 Military Institute of Engineer Technology, Obornicka 136 Str., 50-961 Wroclaw, Poland; dysz@witi.wroc.pl (K.D.); przybyl@witi.wroc.pl (W.P.); 2 Institute of Physics, Jagiellonian University, Prof. S. Lojasiewicza 11, 30-348 Krakow, Poland; monika.marzec@uj.edu.pl; 3 Faculty of Energy and Fuels, AGH University of Krakow, Al. A. Mickiewicza 30, 30-059 Krakow, Poland; cichykac@agh.edu.pl (K.C.); xi@agh.edu.pl (K.Ś.); 4 AGH Centre of Energy, AGH University of Krakow, Ul. Czarnowiejska 36, 30-054 Krakow, Poland

**Keywords:** imine, azomethine, non-covalent interactions, hole-transporting material, thermographic camera

## Abstract

This paper proposes molecular and supramolecular concepts for potential application in perovskite solar cells. New air-stable symmetrical imine, with thiadiazole moieties PPL2: (5E,6E)-N2,N5-bis(4-(diphenylamino)benzylidene)-1,3,4-thiadiazole-2,5-diamine), as a hole-transporting material was synthesised in a single-step reaction, starting with commercially available and relatively inexpensive reagents, resulting in a reduction in the cost of the final product compared to Spiro-OMeTAD. Moreover, camphorsulfonic acid (CSA) in both enantiomeric forms was used to change the HOMO-LUMO levels and electric properties of the investigated imine-forming complexes. Electric, optical, thermal, and structural studies of the imine and its complexes with CSA were carried out to characterise the new material. Imine and imine/CSA complexes were also characterised in depth by the proton Nuclear Magnetic Resonance ^1^H NMR method. The position of nitrogen in the thidiazole ring influences the basicity of donor centres, which results in protonation in the imine bond. Simple devices of ITO/imine (with or without CSA(−) or CSA(+))/Ag/ITO architecture were constructed, and a thermographic camera was used to find the defects in the created devices. Electric behaviour was also studied to demonstrate conductivity properties under the forward current. Finally, the electrical properties of imine and its protonated form with CSA were compared with Spiro-OMeTAD. In general, the analysis of thermal images showed a very similar response of the samples to the applied potential in terms of the homogeneity of the formed organic layer. The TGA analysis showed that the investigated imine exhibits good thermal stability in air and argon atmospheres.

## 1. Introduction

The main disadvantage of new low-molecular materials as new hole-transporting materials (HTMs) developed so far is that they are synthesised via cross-coupling reactions that require transition metal catalysts, inert reaction conditions, and extensive product purification [1]. This makes perovskite solar cells still quite expensive, and it is difficult to scale up their production and application as building materials in photovoltaics (BIPV). However, it should be stressed that while perovskite material is rather cheap, the most common, commercially available hole-transporting material, abbreviated as Spiro-OMeTAD, is several times more expensive than gold or platinum [1,2]. Currently, diphenyl-based HTMs such as Spiro-OMeTAD utilise the Buchwald–Hartwig amination. They require a palladium catalyst, but the amination chemistry requires bromide and a secondary amine which are easily synthesised from commercially available sources. In addition, the amount of palladium catalyst is less than 1 wt% in the reaction, which is not very expensive compared to the reactant and reagent. However, the main problems with Spiro-OMeTAD are the hysteresis effect and its stability in the air. Recently, an HTM in perovskite cells has been designed based on organic and inorganic materials, e.g., poly(3-hexylthiophene): P3HT; poly(3,4-ethylenedioxythiophene): PEDOT; NiO; CuO; CuCrO_2_; CuGaO_2_ MoO_3_; VO_x_; WO_x_; or graphene oxide [3]. It should be emphasised here that both low-molecular compounds and polymers are proposed as HTMs, for example, low-molecular compounds based on triphenylamine (TPA), e.g., [1,4,5,6,7,8,9,10,11,12,13,14]; TPA units with thiophene and phenylene or vinylene–thiophene units as the core [11]; or amide-based small molecules with TPA units [12]. Moreover, imines were also tested for use as HTMs for perovskite cells by Petrus [1,10,15] and Bogdanowicz et al. [15]. Analysing the chemical structure of imines, the influence of both the core and substituents on the obtained photovoltaic parameters of both perovskite cells and organic solar cells was found [1,10,16,17]. In our previous work [15], we investigated the deep photovoltaic properties of 4,4′-((1E,1′E)-((1,2,4-thiadiazole-3,5-diyl)bis(azaneylylidene))bis(methaneylylidene))bis(N,N-di-p-tolylaniline) (bTAThDaz) for use in perovskite solar cells. We compared the selected properties of imine bTAThDaz with those of Spiro-OMeTAD. For the constructed perovskite solar cells with this imine as the HTM, we observed power conversion efficiency PCE = 14.4%. Moreover, the constructed device with imine was time stable and exhibited a negligible hysteresis effect. However, perovskite cell design still relies heavily on the addition of compounds such as tert-butylpyridine (TBP) and lithium bis(trisfluoromethylsulfonyl)imide (Li-TFSI and tris(2-(1H-pyrazol-1-yl)pyridine)cobalt(II)bis(hexafluorophosphate) (FK102) as co-dopants to improve cell efficiency, stability, and lifetime.

Herein, we report the synthesis and characterisations of new symmetrical imine and its complexes with camphorsulfonic acid (CSA) in both enantiomeric forms. We applied the molecular and supramolecular engineering concepts towards designing new imine and its complexes with CSA as novel materials that could meet the required expectations and could be considered for perovskite solar cell construction. In the screening process, imine and imine/CSA complexes were characterised by ^1^H NMR, UV–Vis and cyclic voltammetry to select the best potential candidate for use in the construction of perovskite solar cells. The behaviour of PPL2 imine, composed of a thiadiazole core with triphenylamine substituents, under protonation conditions with 10-camphorosulfonic acid was described for the first time. In our studies, a thermographic camera was used to detect the location of defects in the created devices and the electric behaviour of imine and its complexes with CSA(+) or CSA(−). Finally, to check the crucial electric properties, the electric and thermal response to forward bias was also studied. The thiadiazole-based materials without transition-metal catalysts seem to be useful, but we must consider the synthetic feasibility. This imination chemistry requires aldehyde and a primary amine. In our case, we used commercially available compounds. In our opinion, the synthesised imine product is cheap because it is only a single-step reaction with only water as a co-product.

## 2. Experimental Section

All chemicals and reagents were used as received from Sigma-Aldrich (St. Louis, MO, USA).

### General Synthesis of Imine

**Figure 1:**
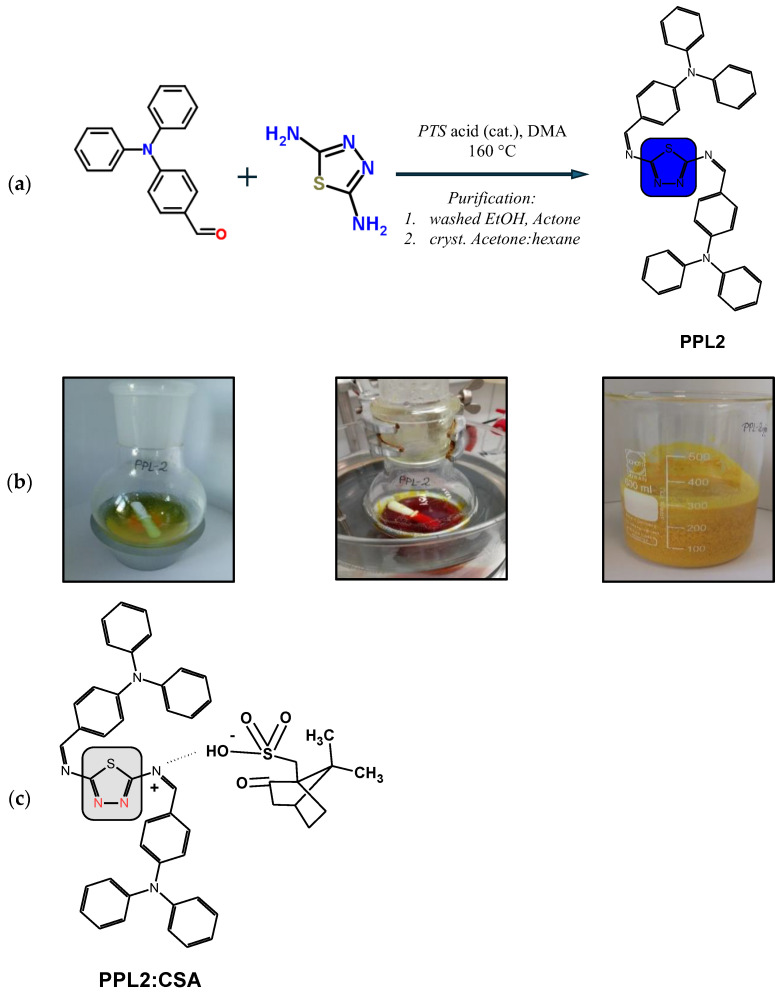
Synthesis scheme of imine PPL2 (**a**); the selected steps of PPL2 synthesis from left to right: before synthesis, after 24 h of synthesis, and after precipitation in water (**b**); scheme of the PPL2 protonation with CSA (**c**).

The imine was obtained using a one-step, high-temperature condensation reaction under reflux equipped with anhydrous CaSO_4_ as the water trap. A single-neck flask with a magnetic stir bar was charged with aldehyde (2 mmol), diamine (1 mmol), *p*-toluenesulfonic acid (PTS), and 12 mL of DMA. The reaction mixture was stirred for 24 h at 160 °C in an oil bath. The raw imine was precipitated in water and collected by filtration. The solid was washed with ethanol and acetone and recrystalised from acetone/hexane. The final imine was dried overnight at 80 °C. Photos of the selected steps of PPL2 synthesis, as an example, are presented in Figure 1b.

PPL2: (5E,6E)-N2,N5-bis(4-(diphenylamino)benzylidene)-1,3,4-thiadiazole-2,5-diamine: yellow powder, Yield: 33%. ^1^H NMR (400 MHz, CDCl_3_), δ [ppm]: 8.56 (2H, s, –HC=N–); 7.65–7.55 (8H, m, arom. –Ph–); 7.4–7.0 (20 H, arom. –Ph). FT-IR (cm^−1^): 3240, 3183 2ν(C=C)_ar_, 3037 ν(CH)_ar_, 2974, 2772, 2438, 2054, 1689 2ω(CCH)_ar_, 1612 ν(HC=N), 1589 ν(C=C)_ar_, 1504 ν(C=C)_ar_, 1487, 1395, 1330, 1312, 1117 δ(CH)_ar_ ip, 1072 δ(CH)_ar_ ip, 1019 δ(CH)_ar_ ip, 1008, 978, 958, 944, 881 δ(ring)_thiadiazole_ oop, 829 ω(CCH)_ar_, 749 δ(ring)_thiadiazole_ oop, 696, 614, 517. (ν—stretching; δ—bending; in plane (ip) modes: ρ—rocking, σ—scissoring; out of plane (oop) modes: ω—wagging, τ—twisting; ar—aromatic), m.p. 191 °C.

The imine was characterised using several techniques. The ^1^H NMR method was applied using deuterated chloroform (CDCl_3_) as a solvent with a Jeol ECZ-400 S (Akishima, Japan) spectrometer (^1^H–400 MHz) with a delay time of 5 s. Measurements were carried out at room temperature on 10–15% (*w*/*v*) sample solutions.

The Fourier transform mid-infrared (MIR) spectra of the imines in the region of 4000–400 cm^−1^ were measured at 2 cm^−1^ resolution with the co-addition of 32 scans on a Nicolet-Nexus spectrometer using the KBr pellet technique.

Differential scanning calorimetry (DSC) measurements were performed using a Perkin Elmer DSC8000 calorimeter (Waltham, MA, USA). The temperature was calibrated on the onsets of the melting points of water and indium. The sample was hermetically sealed in aluminium pans of 30 µL. Measurements were conducted during cooling/heating at a rate equal to 10 °C/min in a nitrogen atmosphere.

Thermogravimetric (TGA) measurements were performed on a TA Q5000 IR thermobalance (New Castle, DE, USA) using Pt holders. The behaviour of the imine was examined under two different atmospheres of synthetic air and Ar (5N). The gas flow was 100 mL min^−1^, supplied directly in the vicinity of the studied sample. Each TGA test included heating in a temperature range of 30–800 °C with a rate of 10 ° min^−1^.

Electrochemical measurements were carried out using a Metrohm Autolab PGSTAT M204 (Barendrecht, The Netherland) potentiostat, and the electrochemical cell contained a glassy carbon electrode (diam. 2 mm), a platinum rod, and Ag/AgCl as working, counter, and reference electrodes, respectively. Potentials were referenced to ferrocene (Fc), which was used as the internal standard. Cyclic voltammetry experiments were conducted in a standard one-compartment cell, in acetonitrile (≥99.9%, Honeywell, Mint Street Charlotte, NC, USA), under argon. Then, 0.2 M Bu_4_NPF_6_ (Alfa Aesar, Tewksbury, MA, USA, 99%) was used as the supporting electrolyte. The concentration of the compound was equal to 1.0 × 10^−6^ mol dm^−3^. The deaeration of the solution was achieved by argon bubbling through the solution for about 15 min prior to the measurement. All electrochemical experiments were carried out at ambient temperature and pressure.

Absorption spectra of imine PPL2 in chloroform were recorded in the range from 400 nm to 800 nm by using a UV–Vis model A360 spectrometer (AOR Instruments, Shanghai, China).

Thermal behaviour was observed using a thermographic camera (VIGOcam v50, VIGO System S.A, Ożarów Mazowiecki, Poland) while applying a bias voltage between 0 and 10 V and using a multichannel potentiostat–galvanostat (PGStat Autolab M101, Metrohm, Schiedam, The Netherlands) connected to a computer. The experiment was programmed as follows: the potential was applied from 0 V to 10 V in steps of 0.5 V for three minutes for each voltage. The current response was recorded during these three-minute intervals, and each step was separated with a 10 s window when the IR image was collected, while the current was still passing through the sample. The work of both the camera and power source was controlled via computer software.

## 3. Results and Discussions

### 3.1. Synthesis and Purification of Imine

New imine denoted as PPL2 (see Figure 1a) was synthesised in a one-step condensation reaction in solution between (4-(N,N-diphenylamino)benzaldehyde) and diamine 2,5-diamino-1,3,4-thiadiazole (PPL2), where water was the main by-product. Therefore, the synthesis path of imine proposed by us was performed in a single-step reaction followed by simple purification, which significantly lowered the production cost of the pure product.

The simplicity of the whole process allows for the recovery of the solvent, i.e., ethanol, acetone, or DMA. Moreover, no expensive catalysts or inorganic compounds are used in the process, and the main by-product, water, is environmentally friendly and can be reused in the process [18].

**Figure 2:**
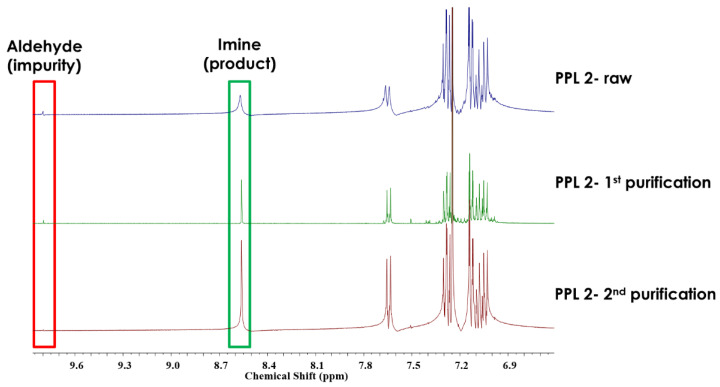
Steps of purifications for PPL2 detected by ^1^H NMR.

Special attention was paid to the purification of the obtained imine by applying various solvents (ethanol, acetone), combined with crystallisation from the acetone–hexane mixture. The purification progress for PPL2 monitored by ^1^H NMR spectroscopy is presented in Figure 2.

After the reaction, the raw product exhibited the presence of unreacted aldehyde (Figure 2). A second round of purification, including recrystallisation from the acetone–hexane, resulted in pure products for all synthesised imines. Progress in the purification process was also controlled by thin-layer chromatography (TLC).

New symmetrical imine was characterised by ^1^H NMR and FTIR spectroscopy. All the experimental data were consistent with the proposed structure. The confirmation of desired product formation was particularly observed in the proton NMR spectra of the investigated imine, where the azomethine proton signal at approximately 8.31–8.56 ppm was observed, as expected. The presence of imine groups was also confirmed by FTIR spectroscopy by the band characteristic stretching deformations of the –HC=N– at 1609–1612 cm^−1^ (see Section 2).

The thermal properties of the investigated imine were characterised by both differential scanning calorimetry (DSC) and thermogravimetric analysis (TGA). Our study showed that the melting point of the imines depended mainly on the thiadiazole core. Imine PPL2 with a 1,3,4-thiadiazole core exhibited a melting point at 191 °C.

**Figure 3:**
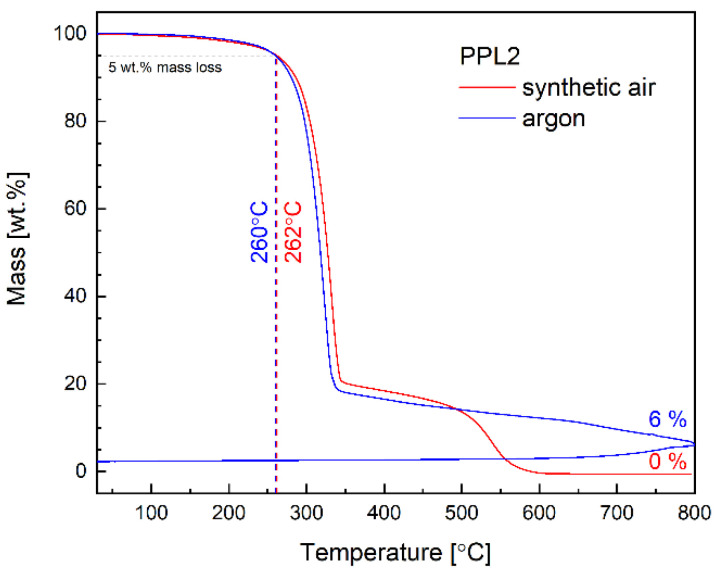
TGA curves of imine studied in air and argon atmosphere.

The results of thermogravimetric analysis (TGA) performed in an argon and air atmosphere at a heating rate of 10 °C/min, from room temperature up to 800 °C, are presented in Figure 3.

The TGA curve of imine indicates two main reaction stages. The initial decomposition based on 5% weight loss occurred, which is usually considered a criterion for assessing thermal stability, within the range of 260–262 °C for PPL2. The TGA analysis suggested that the investigated imine exhibits good thermal stability in air and argon atmospheres. Some differences in the amount of carbonised residue were found depending on the atmosphere applied (see Figure 3). Almost no weight changes occurred below 80 °C, indicating a lack of influence of the adsorbed H_2_O/CO_2_. The initial weight loss was smooth and gradual.

### 3.2. Imine Protonation with Camphorosulfonic Acid Enantiomers

In our previous study, it was found that polyazomethine with triphenylamine and carbazole moieties doped with camphorsulfonic acid (CSA), as an electrical p-dopant, showed improvements in the electrochromic properties as a result of imine bond protonation [19]. Moreover, Lee et al. [20] used CSA in a mixture with polyaniline, as a hole-transporting layer in inverted perovskite solar cells, due to its moisture resistance and flexibility. Here, new imine is proposed to be used as a chromogenic sensor to check the optical and electrical properties of HTM via protonation with enantiomeric forms of CSA ((1S)-(+)-10- or (1R)-(−)-10-), abbreviated in this paper as CSA(+) and CSA(−)). The process of self-organisation (acid–base interactions) facilitates the formation of ordered nanoscale structures and further allows the elaboration of responsive materials towards perovskite solar cells. The scheme of new imine PPL2 protonated with CSA is presented in Figure 1c.

In the first step, the influence of the creation of acid–base interaction between the SO_3_H group in CSA and nitrogen atoms in imine was established by ^1^H NMR spectroscopy. Then, ^1^H NMR studies were performed to obtain an insight into the influence of the presence of both enantiomeric forms of 10-camphorosulfonic acid on imine. The solutions of imine and acid dopant in CDCl_3_ were prepared in an equimolar ratio to unveil the protonation centre preferred in the imine molecule.

**Figure 4:**
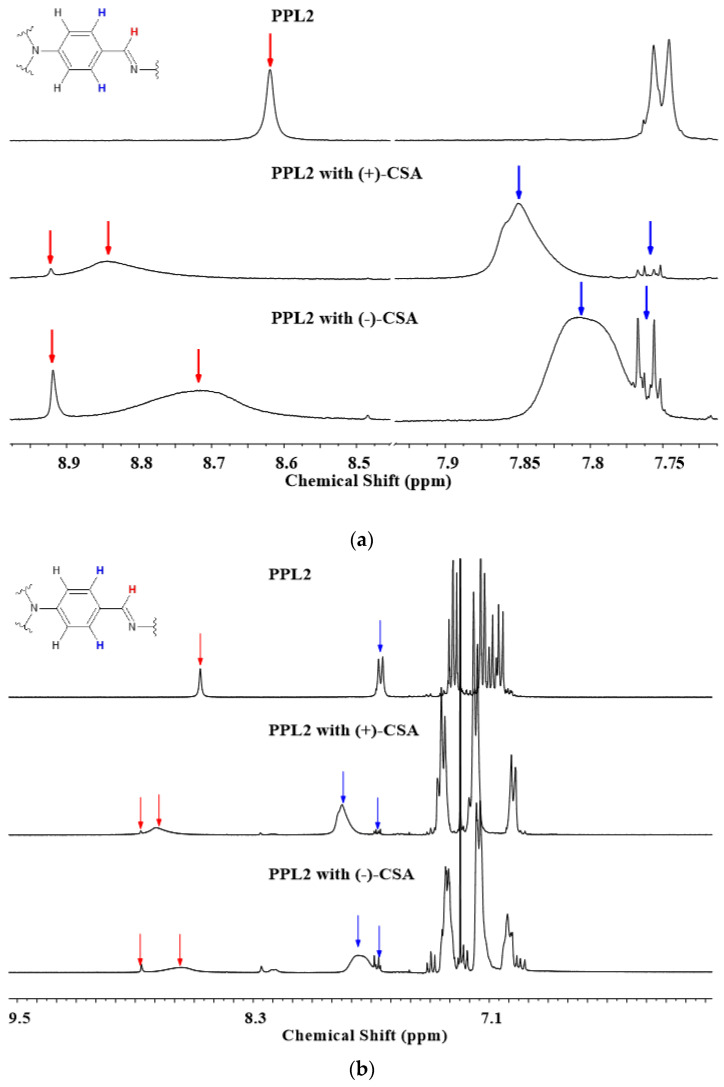
The ^1^H NMR spectra of neat PPL2 and its complexes with CSA(+) and CSA(−) in the full spectral range (**a**) and the onset of signals influenced by the presence of CSA in 1:1 molar ratio in CDCl_3_ (**b**).

The following difference in ^1^H spectra was revealed for imine PPL2 protonated with CSA(+) and CSA(−): the signal of the imine proton (8.6 ppm) shifted towards the lower field and split into two signals: 8.7 ppm and 8.9 ppm for the mixture with CSA(−) and 8.8 ppm and 8.9 ppm for the mixture with CSA(+) (see Figure 4b). The first signal was very broad for both enantiomeric forms, whereas the other was narrow. This suggests that protonated nitrogen resulted in the broadening of the imine proton caused by the screening effect of CSA molecules, whereas the symmetric proton from the other imine group became more exposed as an effect of electron withdrawal from the double bond. Also, the phenyl protons of triphenylamine closer to the imine bond were affected by the protonation, giving the same effect as described for the imine proton: they split into two signals and shifted towards the lower field from 7.65 ppm (doublet) to 7.67 ppm (doublet) and a broad singlet at 7.76 ppm and 7.85 ppm for CSA(−) and CSA(+), respectively. The results indicate that protonation occurred on the imine nitrogen, causing the shifting and splitting of signals, since only one imine nitrogen could be protonated because of the ratio of two imine nitrogen/one CSA. For the PPL2:CSA mixture, those findings align very well with the UV–Vis results, where an additional maxima can be observed for mixtures, which indicates the presence of monoprotonated imine nitrogen. Zubert et al. [21] mentioned that for aminoisooxazoles, protonation unambiguously depends on the substituent position; however, it also depends on the state of the sample. Furthermore, in this study, for 2-amino-1,3,4-thaidiazoles derivatives, the protonation started from the amine group.

In the literature, Serban et al. discussed the isosterism influence on biological activity because of a different arrangement of heteroatoms in the thiadiazole ring, which causes the difference in the behaviour of the molecule; 1,2,4-thiadiazole has some similar biological behaviour to pyrimidine, whereas 1,3,4-thiadiazole is like pyridazine [22]. In our understanding, the position of nitrogen in the thiadiazole ring has an influence on the basicity of donor centres, which results in the protonation of the imine bond of PPL2.

Moreover, as shown later in the paper, the influence of acid–base interactions occurring between the SO_3_H group in CSA and nitrogen atoms in imine on the electrical, thermal, and optical properties of imine was examined using CV and UV–Vis methods.

### 3.3. Optical and Electrochemical Studies

The electrochemical oxidation and reduction onset potentials were used to estimate the HOMO and LUMO energies (or rather, ionisation potentials (IPs) and electron affinities) of the material studied, assuming the IP of the reference ferrocene to be −5.1 eV [23]. The HOMO-LUMO levels obtained together with the electrochemical energy band gap (Eg^CV^) are presented in Table 1.

Generally, the addition of an equimolar portion of CSA increased the energy gap (E_g_) compared to the pure imine. The CSA molecule influenced the E_g_ by increasing its value, maximally, of 0.08 eV. Apart from the offset redox process, another reduction process was revealed with maxima at −0.9 V, whereas the oxidation processes were detected at 0.03 V, 0.27 V, and 0.94 V, where the last one was used to designate the onset oxidation process. For mixtures with CSA, the right-handed enantiomer CSA(+) caused the exhibition of only one oxidation and one reduction process, moving the oxidation onset from 0.76 V to 0.70 V and the reduction offset from −2.19 V to −2.31 V. The addition of the left-handed enantiomer CSA(−) did not reduce the number of oxidation processes and only shifted their maxima from 0.03, 0.27, and 0.94 to −0.67, 0.33, and 0.90, respectively. For the reduction, the CSA(+) influenced the reduction offset of about 0.12V.

In all the cases, the lowest original cathodic peak came from thiadiazole due to its strong aromaticity [24], and two more noticeable cathodic peaks could be assigned to the imine bond. The acidic additive, which protonated the imine bond as described above in the NMR study, significantly affected the electrochemical properties.

The conductivity values for all tested layers based on the current–voltage results obtained during the thermal image registration are organised in decreasing order: PPL2:CSA(−) > PPL2 > PPL2:CSA(+). The conductivity data demonstrated a tendency, with the addition of the CSA(−), to improve the conductivity of the organic layer in comparison to the neat imine, whereas the addition of CSA(+) gave the opposite effect. When assessing the influence of the thiadiazole ring on the conductivity values, the 1,2,4 position showed better conductivity by approximately 20% than the 1,3,4 position. The addition of CSA gave a change in the conductivity value by pprox. 0.6 × 10^−8^ S/cm, improving with the CSA(−) or reducing with the CSA(+) the value of PPL2.

**Figure 5:**
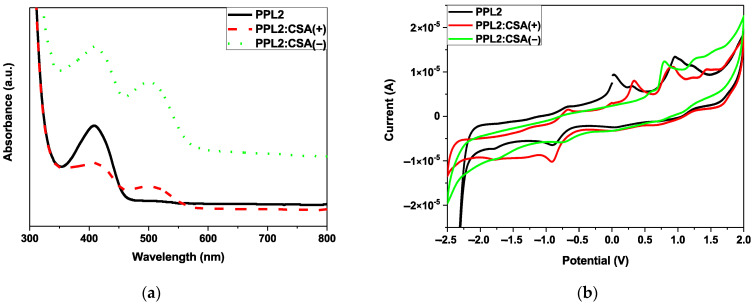
UV–Vis (**a**) and CV (**b**) spectra of imine PPL2 in undoped and protonated form with CSA(+) and CSA(−) in chloroform solution.

The optical properties of chloroform solutions of imine in neutral and protonated form investigated by UV–Vis absorption spectroscopy are summarised in Table 1. Imine PPL2 contained 1,3,4-thiadiazole as a core, and only one absorption maximum was observed at 410 nm. In the case of doped PPL2, two maxima were revealed at 410 nm and 502 nm. These results confirm the bathochromic effect of the acidic dopant present in the equimolar part of the imine solution. The optical and electrochemical properties of PPL2 in doped and undoped form are presented in Figure 5.

### 3.4. Thermal and Electrical Properties

To investigate defects in organic photovoltaics, various microscopic techniques can be applied, e.g., atomic force (AFM), scanning electron (SEM), or transmission electron (TEM); however, thermal imaging is a fast, simple, and low-cost method for detecting defects [25,26,27,28,29]. In our studies, a thermographic camera was used for the first time to find the location of defects in the fabricated simple devices and check the electrical behaviour of imine and its complexes with CSA.

**Figure 6:**
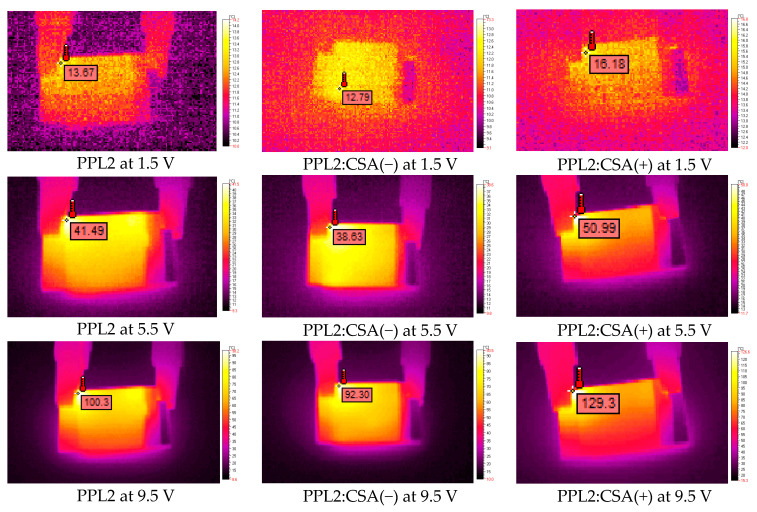
IR images obtained for constructed devices at 1.5 V, 5.5 V, and 9.5 V containing PPL2 and their protonated form with CSA(+) and CSA(−).

Devices with the following architecture were thermally imaged: ITO/PPL2 (with or without CSA(−) or CSA(+))/Ag/ITO, where PPL2 imine layers with or without CSA were deposited by spin coating on ITO conductive glass. The results are shown in Figure 6. CSA was always in an equimolar ratio to the PPL2 compound. In general, the analysis of thermal images showed a very similar response of the samples to the applied potential in terms of the homogeneity of the formed organic layer. Also, it was noticed that all organic layers behave as conductors, exhibiting a decrease in resistance with increasing potential. As a secondary effect of the electrical conductivity, a temperature increase was observed (see Figure 6).

**Figure 7:**
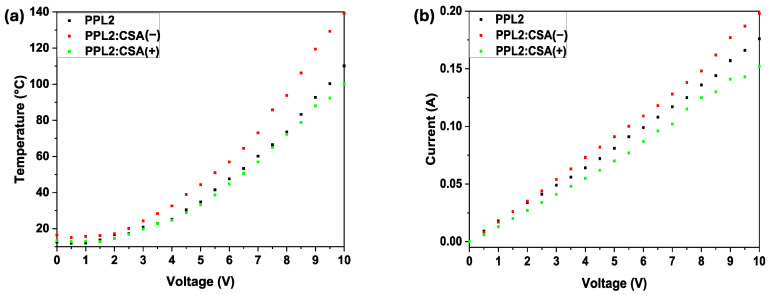
Temperature and applied potential correlation (**a**) and current flow and applied potential correlation (**b**) for constructed devices containing neat PPL2 and its protonated form with CSA.

Imine PPL2, as a pure compound, withstood high temperatures, in some cases even up to 110 °C. The resistance on the 1 cm^2^ layer ranged between 50 and 60 Ω. When assessing the addition of both enantiomeric forms of 10-camphorsulfonic acid, it was observed that the mixture with CSA(−) showed a decrease in resistance value by 6.30 Ω, while CSA(+) caused the opposite effect, causing an increase in resistance value by 6.62 Ω for the mixture with PPL2. The addition of the acidic dopant increased the thermal resistance, in some cases up to almost 140 °C. The dependence of temperature and current on various applied potentials is shown in Figure 7 for neat PPL2 and its protonated form.

The intention behind placing the IR images was to demonstrate the temperature distribution during the increment in applied potential. The maximum temperature observed for each sample during the experiment is displayed in the temperature–voltage plot. As can be observed at the highest registered potentials (9.5 V and 10.0 V), the PPL2:CSA(−) demonstrated stability at the highest observed temperature. The reason for observing such high temperatures is related directly to the fact that this mixture had the highest electrical conductivity, which was proportional to the registered maximum temperature.

Finally, we compared the results obtained by the thermographic camera for the fabricated device in this work with the device containing Spiro-OMeTAD that we had previously constructed [15]. The thermal imaging for the device containing Spiro-OMeTAD as an organic layer registered only up to 6.5 V with the same setup, which resulted in a mostly uniform temperature distribution with noticeable heat centres at the edges close to both electrodes, in contrast to devices containing the PPL2 compound, where the highest temperature centres were observed only close to the positive electrode or in the central part for the neat PPL2 layer and mixtures of PPL2 with CSA(+) or CSA(−). This might suggest that the most thermally consuming process was the charge transfer between two interfaces of the organic layer/electrode, whereas the conduction process within the thin layer seemed more uniform. It is worth mentioning the fact that spiro-OMeTAD showed lower resistance equal to 24.05 Ω—nearly double the value of doped mixtures. For the 6.5 V potential, the highest registered result for spiro-OMeTAD, the temperature was higher by approx. 30 °C when compared to the doped PPL2 [15]. The biggest disadvantage of the commercial material is the existence of local heat maxima located close to both metallic electrodes.

To summarise, bearing in mind that charge transport in organic semiconductors typically occurs via a hopping mechanism, it is evident that especially CSA(−) has a positive effect on the charge transfer observed in the PPL2 compound. The presence in the structure of an electron-rich five-member ring linked via an imine bond to a triphenylamine facilitated the possibility of forming a stable interaction between CSA and the imine bond without changing the original optical absorption band, but even expanding in a bathochromic direction allowed the absorption of more energetic photons. The band split of the main absorption band into two signals, in the proportion of 2/3 to 1/3, allowed the possibility of increasing the amount of the doped organic semiconductor in the thin layer, further improving photon harvesting. It is worth mentioning that the HOMO-LUMO levels did not change significantly in comparison to the undoped material, even, in some cases, reducing the number of observed redox processes, hence improving electrochemical stability. In this study, we focused on the charge transfer aspect from the electric conductivity point, registering the thermal self-heating process. The conductivity of the organic layer changed with the addition of acid dopant, showing an improvement for CSA(−) and a decrease for CSA(+). It was evident that only the isomer CSA(−) seemed sterically compatible with the molecular structure of imine PPL2, resulting in overall improvements in all major properties.

Taking into account the similarities to bTAThDaz described in the literature [15], it is likely that PPL2, especially in the doped form with CSA(−), might have very similar properties when used as a substitute for spiro-OMeTAD. Based on the neglectable differences in the HOMO-LUMO energy values, conductivity, thermal stability, and similar molecular structure, it is likely to perform similarly in application as an HTM in perovskite solar cells.

### 3.5. Durability at Ambient Conditions and Prices of PPL2

It needs to be stressed that the durability at ambient conditions for the imine layer was observed during thermo-electrical studies, where the sample was not hermetically sealed from the ambient conditions. The TGA studies presented in this paper are the standard procedures to perform for new materials. As it was described, the unsealed samples showed resistance to ambient conditions even at elevated temperatures.

**Figure 8:**
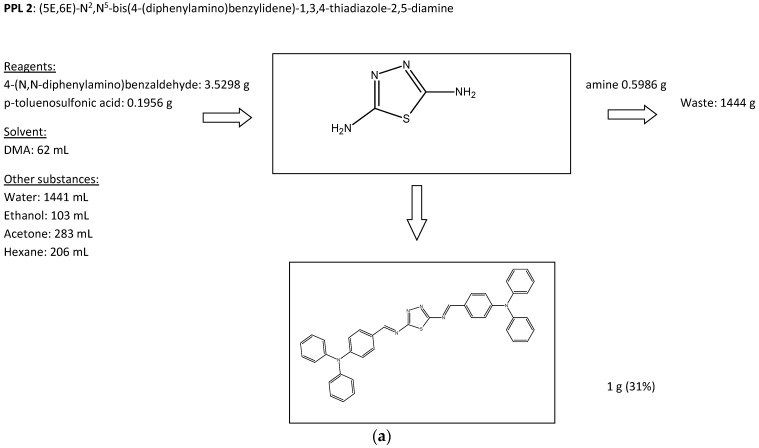

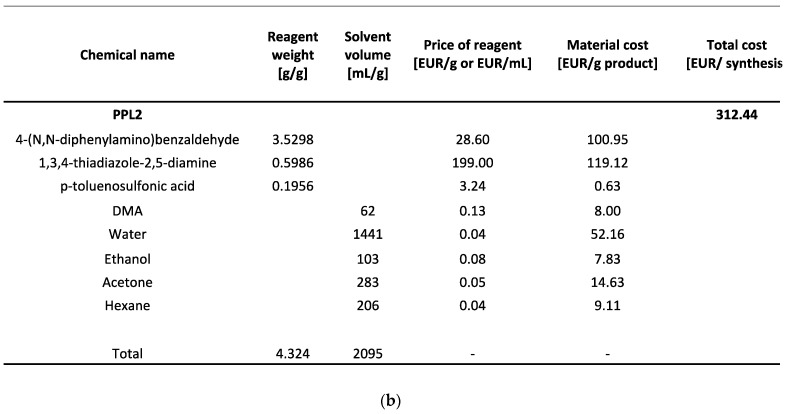
The calculated prices of synthesised imine PPL2. (**a**) flow charts; (**b**) survey of the estimated chemical synthesis cost and waste streams.

Finally, we calculated the prices of synthesised imine, as is presented in Figure 8. The presented prices represent the cost of the laboratory-scale process, which in comparison to commercial spiro-OMeTAD, has the same price. What is known from the literature regarding the scaling-up processes from the laboratory to the industrial level is that the cost is typically 15% of the laboratory cost. Taking this into account, the cost of the PPL2 compound could be roughly 10 times lower than that of spiro-OMeTAD.

## 4. Conclusions

We have presented a new low-molecular hole-transporting material based on TPA units and thiadizole moieties and its complexes with CSA as candidates as hole-transporting layers for perovskite solar cells. Our research can be summarised as follows:

- The synthesis of imine can be performed in a one-step reaction in solution followed by simple purification, which significantly lowers the cost production of the pure product;
- The simplicity of the process allows for the recovery of the used solvent to be reused again in other processes;
- The presence of imine bonds next to the thiadiazole ring resulted in the selective protonation of the structure, namely, the protonation occurs on imine nitrogen;
- The spectral characteristics of PPL2 are almost identical to the spectra of the spiro-OMeTAD commercial compound;
- The addition of the acidic dopant increases the thermal resistance of the synthesised imine PPL2, in some cases up to almost 140 °C.

Considering all the reported results, the designed and synthesised new imine PPL2 appears to be a promising material for use as a conductive layer, preferably HTL, in perovskite solar cells. The techniques presented in this paper for the characterisation of organic semiconductors in the form of thin layers show potential as a screening method for assessing the basic properties of a material prior to the construction of solar cells. In our previous work, we used 4-(di-p-tolylamino)benzaldehyde) and 1,2,4-thiadiazole-3,5-diamine [15] instead of 4-(diphenylamino)benzaldehyde, which was applied in the present work. For the constructed perovskite solar cells with the architecture glass/FTO/TiO_2_/perovskite/imine bTAThDaz/Ag, we obtained photovoltaic parameters such as the power conversion efficiency PCE = 14.4%, the open circuit voltage Voc = 0.90 V, the short-circuit current Jsc = 22.96 mA/cm^2^, and the fill factor FF = 0.70. An extensive study of perovskite solar cells containing imine PPL2 in their structure needs to be carried out and will be the subject of our work in the future.

## Figures and Tables

**Table 1:** Electrochemical (CV) and optical (UV–Vis) properties of imine PPL2 and its protonated form with CSA.

Sample	E_ox_^onset^ [V]	E_red_^offset^ [V]	HOMO [eV]	LUMO [eV]	Eg^CV^ [eV]	λ_abs_ [nm] in CHCl_3_	Conductivity S/cm
PPL2	0.74	−2.19	−5.19	−2.38	2.81	410	4.61 × 10^−8^
PPL2:CSA(+)	0.68	−2.24	−5.13	−2.33	2.80	414, 510	4.16 × 10^−8^
PPL2:CSA(−)	0.70	−2.31	−5.15	−2.26	2.89	412, 504	5.21 × 10^−8^

## Data Availability

Data are contained within the article.
